# Highly efficient photocatalytic performance of BiI/Bi_2_WO_6_ for degradation of tetracycline hydrochloride in an aqueous phase

**DOI:** 10.1039/d0ra01811b

**Published:** 2020-03-25

**Authors:** Keyi Zhou, Jianjiang Lu, Yujun Yan, Chengyu Zhang, Yijin Qiu, Wanjie Li

**Affiliations:** School of Chemistry and Chemical Engineering, Shihezi University Shihezi 832003 China lujianjiang_xj@163.com yyj_tea@shzu.edu.cn; Key Laboratory for Environmental Monitoring and Pollutant Control of Xinjiang Production and Construction Corps, Shihezi University Shihezi 832003 China; Environmental Monitoring Station of the First Division of Xinjiang Production and Construction Corps China

## Abstract

A series of novel BiI/Bi_2_WO_6_ nanosheets was successfully synthesized using a simple and efficient one-step hydrothermal method; the obtained specimens were subsequently characterized using X-ray diffraction, scanning electron microscopy, transmission electron microscopy, ultraviolet-visible spectrophotometry, X-ray photoelectron spectroscopy, N_2_ adsorption/desorption isotherms, Raman spectroscopy, ultraviolet-visible spectroscopy, Fourier-transform infrared spectroscopy, photoluminescence, and electronic impedance spectroscopy testing. The results indicated that the photocatalytic performance of the BiI/Bi_2_WO_6_ composites for the degradation of tetracycline hydrochloride (TC) from aqueous media under visible light irradiation (*λ* > 420 nm) was higher than that of pure Bi_2_WO_6_. The 0.8I-BiI/BWO composite (where 0.8 is the I : W molar ratio) presented the best photocatalytic performance of all analyzed specimens, and was able to degrade approximately 90% of the TC in 80 min. In addition, radical-capture experiments have demonstrated that superoxide anion radicals and hydroxyl radicals were the main active species for degrading organic pollutants, and a photocatalytic mechanism for the BiI/Bi_2_WO_6_ system was proposed. This study not only provides a method for the simple preparation of BiI/Bi_2_WO_6_, but could also present important implications for ecological risk management and prevention against antibiotic pollution.

## Abstract 2

This study provides a method for the simple preparation of BiI/Bi_2_WO_6_, and present important implications for prevention against antibiotic pollution.

## Graphical abstract

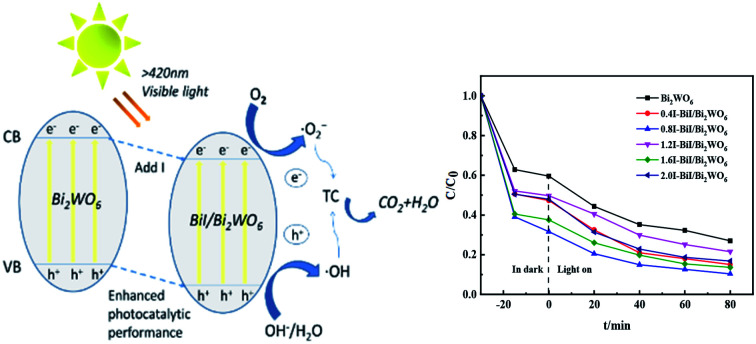

## 1. Introduction

Recently, the use of antibiotics has continued to increase, and antibiotics have become some of the most widely used and commonly used drugs worldwide. Antibiotic pollutants are frequently detected in water sources, and therefore, they have become a new class of environmental pollutant.^1–3^ Tetracycline hydrochloride (TC), which is a typical antibiotic, is widely distributed in water bodies, and barely decomposes naturally owing to its antibacterial and chemical stability. Moreover, its metabolites enter the environment and negatively affect the quality of water sources and the safety of ecosystems.^4,5^ Furthermore, owing to its large amount and high frequency of use, TC is continuously discharged into the ecological environment, and thus leads to its “false” persistence in the environment.^6^ Therefore, reducing TC pollution is a significant concern.

Compared with traditional biochemical and physicochemical methods, photocatalytic technology is a new water treatment technology that has been developed recently for the effective removal of antibiotics.^7,8^ Photocatalytic materials present great potential for converting solar energy into chemical energy; moreover, they can be used as green materials for the effective removal of various organic pollutants during water treatment, which has attracted much attention in environmental restoration research.^9,10^ A large number of semiconductor photocatalysts with different functional compositions that could efficiently use solar energy have been developed to date. Of them, bismuth tungstate (Bi_2_WO_6_) is the simplest Aurivillius-type oxide that features WO_6_ perovskite structure sheet, and is a typical semiconductor photocatalyst.^11,12^ The band gap width of Bi_2_WO_6_ (∼2.8 eV) is narrower than that of TiO_2_, which is a traditional photocatalyst, and therefore, Bi_2_WO_6_ could be excited using visible light and could be used as a new photocatalytic material with visible light response ability.^13–16^ Therefore, Bi_2_WO_6_ photocatalytic materials present potential practical value for environmental purification and new energy development.

However, studies have demonstrated that the molecular weight of Bi_2_WO_6_ is large, and the commonly obtained Bi_2_WO_6_ grains are prone to shortcomings, such as the recombination of photogenerated electron–hole pairs, small specific surface area, and insufficient number of active sites, which affect their catalytic performance and greatly limit their application range.^17–19^ To overcome these limitations, many feasible studies have been carried out, such as ion doping,^20^ surface metal deposition,^21^ morphology control,^22^ and compounding of semiconductor photocatalysts.^23^ Of these strategies, non-metallic element doping enables the formation of open structures and simultaneously generates an indirect transition mode, which facilitates the efficient separation and charge transfer of the electron–hole pairs, and therefore, could improve the photocatalytic activity of Bi_2_WO_6_. It has been reported that F doping narrows the band gap and enhances the visible light photocatalytic activity of Bi_2_WO_6_ for the degradation of rhodamine B.^24^ Zhou *et al.* revealed that Br doping promoted the charge transfer and prevented the photogenerated electrons and holes of Bi_2_WO_6_ from recombining.^25^ Furthermore, the band gap of Bi_2_WO_6_ was narrowed by the insertion of I^−^ ions into the Bi_2_WO_6_ interlayer and the enhanced the photoactivity of Bi_2_WO_6_ for water purification under visible light irradiation.^26^ Zhang *et al.* prepared an I-doped Bi_2_WO_6_ (I-BWO) photocatalyst using the hydrothermal method and determined that the multivalent iodine species, including I^0^ and I^−^, were co-adsorbed on the defective surface of Bi_2_WO_6_ to form I-BWO.^27^ Therefore, a stable heterostructure was established by regulating the interactions between the amount of I dopant and Bi_2_WO_6_ photocatalyst in this study, which could significantly improve the photocatalytic activity of Bi_2_WO_6_ toward the degradation of pollutants under visible light irradiation.

Consequently, in this study, we prepared BiI/BWO composites with different molar ratios using the one-pot hydrothermal reaction method. Our results demonstrated that this is a simple method for preparing highly crystalline products. Moreover, the photocatalytic activity of the obtained BiI/BWO composites for the photocatalytic degradation of TC in water media under visible light irradiation was significantly enhanced compared with that of pure Bi_2_WO_6_. This research is not only important for ecological risk management and preventing antibiotic pollution, but could also enrich the environmental photochemistry theories.

## 2. Experimental

### 2.1 Materials and reagents

Tetracycline hydrochloride was purchased from Dr. Ehrenstorfer GmbH (Augsburg, Germany). Sodium tungstate dihydrate (Na_2_WO_4_·2H_2_O), bismuth(iii) nitrate pentahydrate (Bi(NO_3_)_3_·5H_2_O), and potassium iodide (KI) were purchased from Sinopharm Chemical Reagent Co., Ltd (Shanghai, China). Isopropanol (IPA), triethanolamine (TEOA), and formic acid (HCOOH) were purchased from Aladdin Biotechnology Co., Ltd (Shanghai, China). Benzoquinone (BQ) and sodium azide (NaN_3_) were purchased from Titan Technology Co., Ltd (Shanghai, China). All chemicals were of analytical grade. Deionized water (18.25 MΩ cm^−1^) was used for all experiments.

### 2.2 Preparation of BiI/BWO photocatalysts

**Fig. 1:**
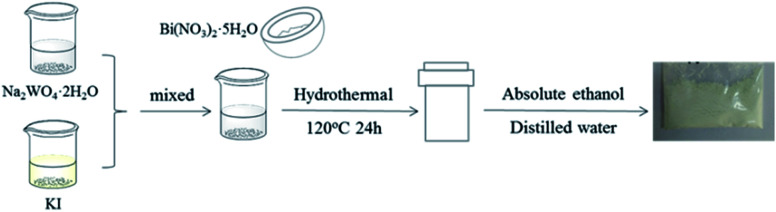
Schematic of catalyst synthesis.

The BiI/BWO samples were synthesized using the hydrothermal method. Typically, 1 mmol Na_2_WO_4_·2H_2_O was added to 80 mL deionized water at room temperature under continuous stirring. Afterward a certain amount of KI was also dissolved in water and the two solutions were mixed together and continuously stirred for 30 min. Next, 2 mmol Bi(NO_3_)_3_·5H_2_O, which was pre-ground, was added to the above solution. After the reaction system was fully stirred, it was transferred to a 100 mL Teflon-lined stainless-steel autoclave and was maintained at 120 °C for 24 h. After the suspensions were naturally cooled to room temperature, they were washed with ethanol and deionized water several times, and were dried at 60 °C overnight. The final samples, which were prepared using 0.4, 0.8, 1.2, 1.6 and 2.0 mmol KI were denoted 0.4I-BiI/BWO, 0.8I-BiI/BWO, 1.2I-BiI/BWO, 1.6I-BiI/BWO, and 2.0I-BiI/BWO, respectively. For comparison, pure Bi_2_WO_6_ was also prepared using the above-mentioned method in the absence of KI. The preparation process is shown in Fig. 1.

### 2.3 Characterization

X-ray powder diffraction (XRD) analysis was carried out using a Bruker AXS, D8 Advance powder diffractometer with Cu Kα radiation (*λ* = 0.15406 nm). The morphology of the specimens was observed using a scanning electron microscopy (SEM; Sigma 300 Zeiss) instrument with the accelerating voltage of 5.0 kV. Transmission electron microscopy (TEM) and high-resolution transmission electron microscopy (HRTEM) measurements were carried out on a JEOL, JEM2100 microscope. Raman spectra were recorded at room temperature using a Renishaw RM2000 system with the laser excitation of 1064 nm. The UV-vis diffuse reflection spectra (DRS) of the specimens were measured on a Shimadzu UV2700/2450 recording spectrophotometer using BaSO_4_ as the reference. A Micromeritics ASAP 2460 analyzer was used to measure the Brunauer–Emmett–Teller (BET) surface areas of the samples at liquid N_2_ temperature. A Thermo Escalab 250xl apparatus with non-monochromated Mg Kα radiation as the excitation source was employed for X-ray photoelectron spectroscopy (XPS) testing. A Nicolet 6700 spectrometer was used to record the Fourier-transform infrared (FT-IR) spectra of the specimens in the wavenumber range of 4000–400 cm^−1^. Photoluminescence (PL) spectra were obtained using an Edinburgh FLS980 fluorescence spectrometer. Electrochemical impedance spectroscopy (EIS) measurements were performed using an Chenhua electrochemical workstation in a three-electrode quartz cell using a mixture of 5 mM K_3_Fe(CN)_6_, K_4_Fe(CN)_6_, and 0.1 M KCl as the electrolyte.

### 2.4 Photocatalytic activity tests

The photocatalytic activity of the photocatalysts for the degradation of TC was evaluated. A 350 W Xe lamp (Nanjing Xujiang) equipped with a 420 nm cutoff filter was used as the simulated sunlight source. The photocatalytic activity tests were carried out in a sealed 50 mL Pyrex glass reactor at room temperature. During a typical experiment, 20 mg photocatalyst was added to 50 mL TC (0.4 g L^−1^) aqueous solution. Prior to irradiation, the suspensions were vigorously stirred for 30 min in the dark to reach adsorption–desorption equilibrium. After a certain time interval, 5 mL liquid was sampled and centrifuged to obtain the supernatant, followed by filtration with a 0.22 μm Millipore filter. The concentrations of TC was determined using a Shimadzu UVmini-1240 UV-vis spectrophotometer by recording its characteristic absorbance at 357 nm, and the photodegradation rates were calculated using eqn (1).

1 Photodegradation rate = (1 − *C*_*t*_/*C*_0_) × 100%

where *C*_0_ and *C*_*t*_ are the initial concentration of TC and its concentration at time *t*, respectively.

### 2.5 Trapping experiments

To identify the dominant species during the photodegradation process and to investigate the photocatalytic mechanism of the photocatalytic process, BQ, IPA, TEOA, NaN_3_, and HCOOH were used as trapping agents for superoxide anion radical, hydroxyl radical, hole, hydroxyl radical and singlet oxygen, and electron (˙O_2_^−^, ˙OH, h^+^, ˙OH + ^1^O_2_, and e^−^), respectively. The experimental procedure was similar with that of the photocatalytic process: an appropriate amount of quencher was suspended in 50 mL TC solution under constant stirring, then 20 mg BiI/BWO photocatalyst was added to the reaction mixture. The detection and calculation methods were the same as those described in Section 2.4.

## 3. Results and discussion

### 3.1 Characterizations of as-prepared BiI/BWO composites

**Fig. 2:**
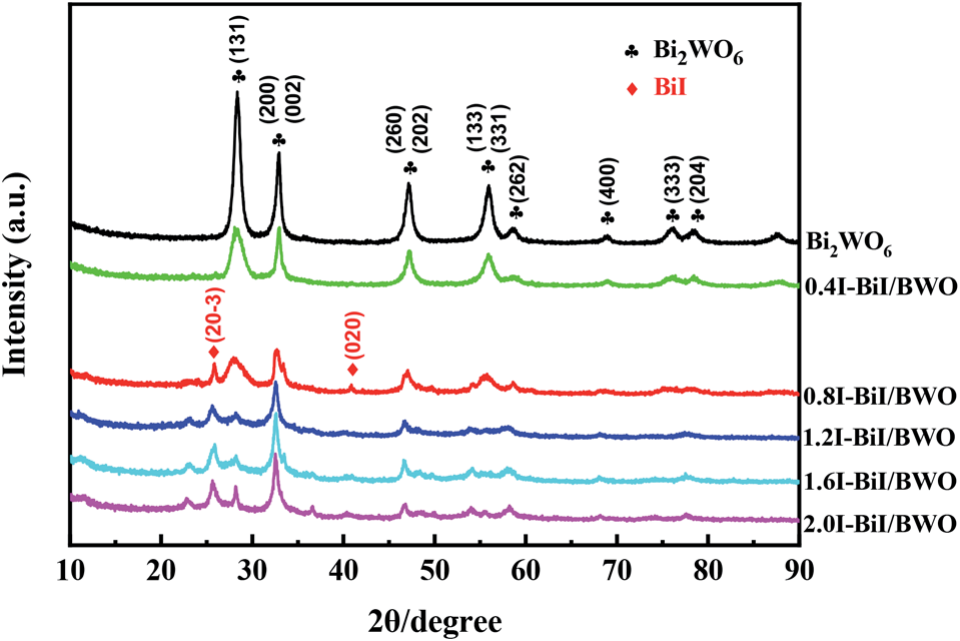
XRD patterns of Bi_2_WO_6_ and BiI/BWO.

The crystal phase and structure of the as-prepared BiI/BWO composites were first examined using XRD measurements. All peaks in the XRD spectrum of Bi_2_WO_6_ (Fig. 2) could be indexed to the orthorhombic phase of Bi_2_WO_6_, which was in good agreement with the previously reported data (JCPDS no. 39-0256).^28^ In addition, all the characteristic diffraction peaks of Bi_2_WO_6_ were observed in the XRD spectra of the BiI/BWO composites, while the diffraction peaks of BiI gradually appeared. However, the peak intensity of the composites decreased as the concentration of KI increased, which indicated that KI could have inhibited the growth of the Bi_2_WO_6_ crystals to a certain extent, but did not affect their growth direction. Moreover, the position of the diffraction peak of Bi_2_WO_6_ shifted slightly toward a lower 2*θ* value, which indicated that the increase in the interplanar spacing of the composites was caused by the interstitial doping of oxygen species.^26^ The distinct diffraction peaks of BiI, which were ascribed to the (20−3) and (020) crystal planes of the 0.8I-BiI/BWO composites were observed in the XRD spectrum of this composite. The peak intensities were high, which indicated that BiI was successfully incorporated into the 0.8I-BiI/BWO composite.

**Fig. 3:**
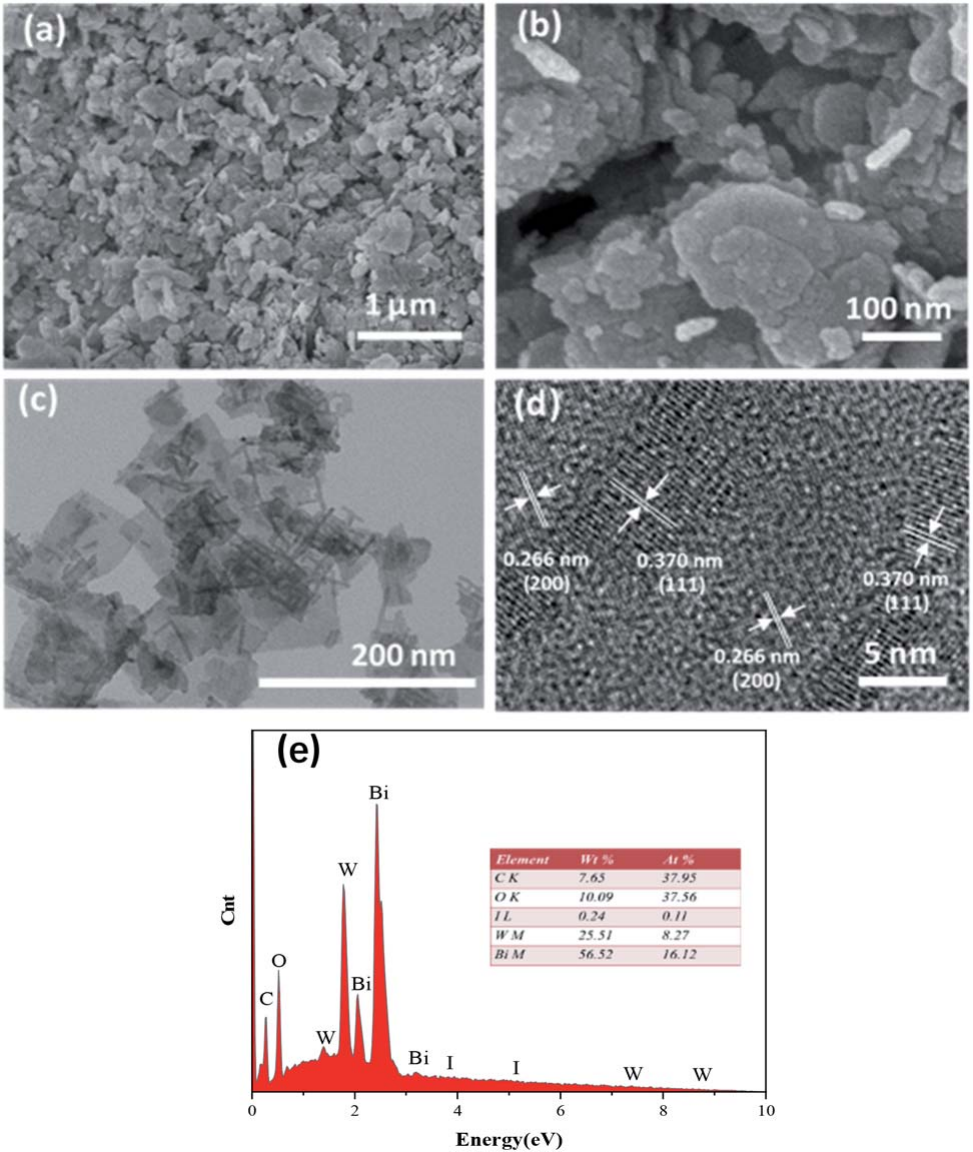
(a and b) SEM image of 0.8I-BiI/BWO, (c) TEM image of 0.8I-BiI/BWO, (d) HRTEM images of 0.8I-BiI/BWO and (e) the EDS mapping of 0.8I-BiI/BWO.

The SEM images of the prepared materials are depicted in Fig. 3(a) and (b), where the flaky morphology of pristine Bi_2_WO_6_ can be observed. Moreover, BiI nanostrips were uniformly distributed on the surface of Bi_2_WO_6_, which demonstrated that BiI was successfully incorporated with Bi_2_WO_6_. More detailed information on the morphology and microstructure of 0.8I-BiI/BWO composites was obtained using TEM and HRTEM, and the images are depicted in Fig. 3(c) and (d), respectively. The HRTEM image of the 0.8I-BiI/BWO nanoplate revealed clear lattice fringes, and the lattice spacings of 0.370 and 0.266 nm corresponded to the (111) and (200) crystal planes of Bi_2_WO_6_, respectively.^26,29^ Additionally, the SEM-energy-dispersive X-ray spectroscopy elemental mappings of 0.8I-BiI/BWO revealed the homogeneous distribution of the component elements (C, O, I, W and Bi) within the composite (Fig. 3(e)).

**Fig. 4:**
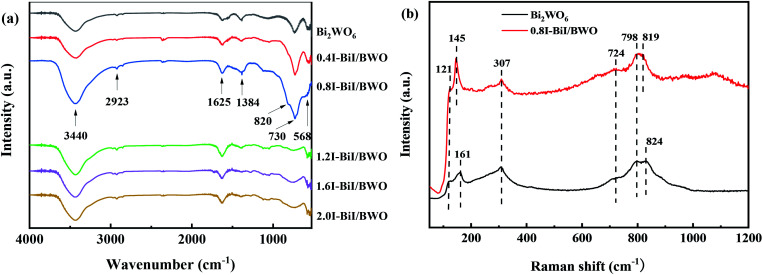
(a) FTIR of Bi_2_WO_6_ and BiI/BWO and (b) Raman spectrum of Bi_2_WO_6_ and 0.8I-BiI/BWO.

The structure of the BiI/BWO composites was further confirmed using FT-IR testing. The absorption bands from 1625 to 3440 cm^−1^ in the FT-IR spectra of Bi_2_WO_6_ and the BiI/BWO composites (Fig. 4(a)) could be ascribed to the stretching vibration of the O–H bonds of water molecules adsorbed on the surface of Bi_2_WO_6_.^13^ The main bands in the range of 500–1400 cm^−1^ were ascribed to the stretching of the W–O (730 cm^−1^) and Bi–O (568 cm^−1^) bonds and the stretching and bending vibration modes of W–O–W (820 and 1384 cm^−1^).^30,31^ Moreover, I doping caused all the stretching vibrations in the FT-IR spectrum of 0.8I-BiI/BWO to shift toward the high frequency region. This indicated that the strong chemical interactions between the I source and Bi_2_WO_6_ was one of the key factors for improving the photocatalytic performance of Bi_2_WO_6_.

Fig. 4(b) illustrates the Raman spectra of pure Bi_2_WO_6_ and the 0.8I-BiI/BWO composite. Several peaks in the range of 600–1000 cm^−1^ were assigned to the stretching of the W–O bonds.^32^ In addition, the intensity of the 724 cm^−1^ peak was ascribed to the vibration of O atoms, and the peaks at 798 and 824 cm^−1^ were assigned to the anti-symmetric and asymmetric vibration of the O–W–O groups, respectively.^33^ The peak at 307 cm^−1^ could be attributed to the translation modes that involved the simultaneous motion of the Bi^3+^ and WO_6_^6−^ ions. The differences between the two samples were not significant, however the peaks at 824 and 121 cm^−1^ in the spectrum of Bi_2_WO_6_ blue shifted. These results indicated that BiI and Bi_2_WO_6_ formed chemical bonds, and the chemical bond energy of Bi_2_WO_6_ in the 0.8I-BiI/BWO composites photocatalyst was different than that of pure Bi_2_WO_6_.^23^

**Fig. 5:**
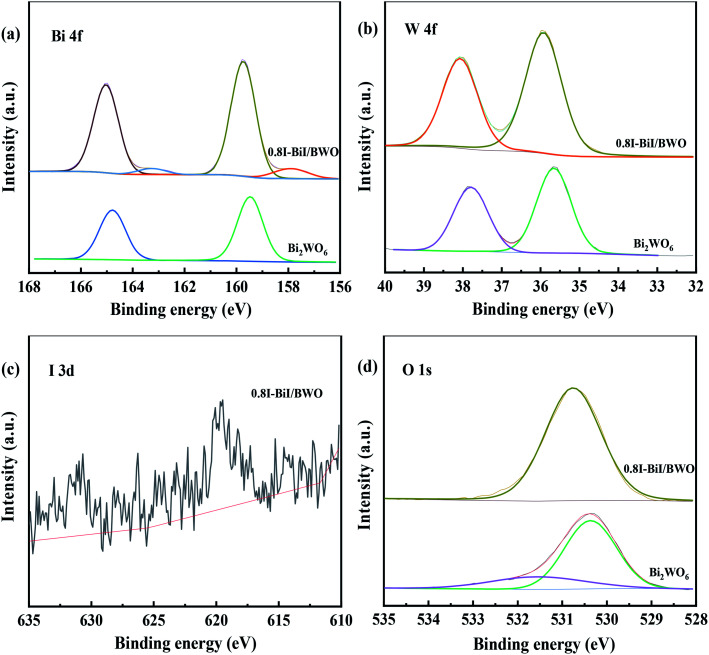
XPS spectra (a) Bi 4f, (b) W 4f, (c) I 3d and (d) O 1s of Bi_2_WO_6_ and 0.8I-BiI/BWO.

The chemical state and surface composition of the as-prepared Bi_2_WO_6_ and 0.8I-BiI/BWO samples were investigated using XPS, and the results are presented in Fig. 5(a)–(d). The XPS profile of Bi was fitted into a doublet that corresponded to Bi 4f_7/2_ and Bi 4f_5/2_. The Bi 4f_7/2_ peak of 0.8I-BiI/BWO consisted of two components, which were attributed to metallic Bi and Bi^3+^.^34^ However, after the addition of KI, the peak of Bi^3+^ in the XPS profile of 0.8I-BiI/BWO was upshifted by 0.2 eV compared with that of Bi^3+^ in the XPS profile of pure Bi_2_WO_6_. Moreover, the W 4f peaks at 35.7 and 37.8 eV, which were ascribed to W 4f_7/2_ and W 4f_5/2_ of the W^6+^ ion in Bi_2_WO_6_ upshifted by approximately 0.2 eV after the addition of the I^−^ ions. Furthermore, the splitting peaks at the binding energies of approximately 630.7 and 619.5 eV in the I 3d XPS profile of 0.8I-BiI/BWO were assigned to I 3d_3/2_ and I 3d_5/2_, respectively.^35^ The asymmetric XPS profile of O 1s (Fig. 5(d)) could be fitted into two contributions: the crystal lattice O of Bi_2_WO_6_ and surface-adsorbed O (˙OH or H_2_O). Unlike the binding energies of Bi 4f and W 4f, that of O 1s of 0.8I-BiI/BWO was increased by approximately 0.3 eV compared with that of pure Bi_2_WO_6_. However, the corresponding XPS profiles of Bi, W, and O of the 0.8I-BiI/BWO composite were shifted compared with those of Bi_2_WO_6_. This could be attributed to the chemical interactions between BiI and Bi_2_WO_6_ and effective electron transfer at the 0.8I-BiI/BWO interface.^36^

**Fig. 6:**
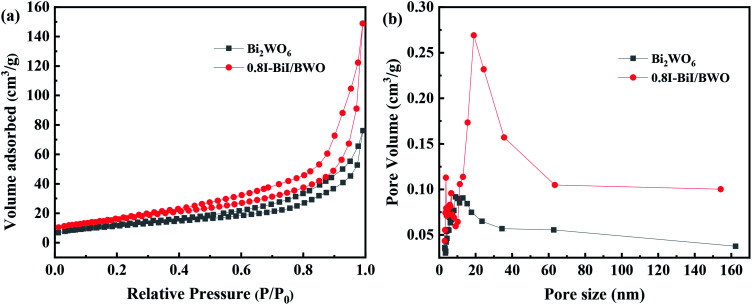
(a) Nitrogen adsorption/desorption isotherms and (b) pore size distribution plots of Bi_2_WO_6_ and 0.8I-BiI/BWO.

Brunauer–Emmett–Teller N_2_ adsorption measurements were conducted to examine the porous nature of the prepared material. The N_2_ adsorption–desorption isotherms of Bi_2_WO_6_ and the 0.8I-BiI/BWO composite catalysts were all type IV isotherms (Fig. 6(a)), which indicated that the catalysts were mesoporous materials.^37^ The BET specific surface areas of Bi_2_WO_6_ and 0.8I-BiI/BWO were 41.101 and 58.101 m^3^ g^−1^, respectively. Therefore, the specific surface area of 0.8I-BiI/BWO was larger than that of Bi_2_WO_6_, which could be attributed to the surface of the 0.8I-BiI/BWO composite being rich in ordered nanoplates. The larger surface area of 0.8I-BiI/BWO could provide more active sites for the adsorption of organic contaminants, which could enhance its photocatalytic activity.^38^ In addition, the pore size of 0.8I-BiI/BWO ranged from 15.0 to 35.0 nm, the maximum pore size was 20 nm, and the pore size distribution was wider than that of pure Bi_2_WO_6_, which indicated that both macropores and mesopores were formed owing to the addition of KI to Bi_2_WO_6_. This could be attributed to the stabilizing effect of KI, which favored the formation of mesopores during the re-synthesis of the composite catalyst. On the one hand, the presence of mesopores favored multilight scattering/reflection, thereby enhanced the capture of excitation light and thus improved photocatalytic activity.^39,40^ On the other hand, the hierarchical porosity composed of macropores and mesopores were conducive to rapid mass transport, resulting in improved photocatalytic activity.^41–45^ The total single-pore pore volume of 0.8I-BiI/BWO was 0.23 cm^3^ g^−1^ at the relative pressure of 0.99141. The extremely high BET surface area and large total pore volume of 0.8I-BiI/BWO confirmed that the nanoparticles presented nanoporous structure.

**Fig. 7:**
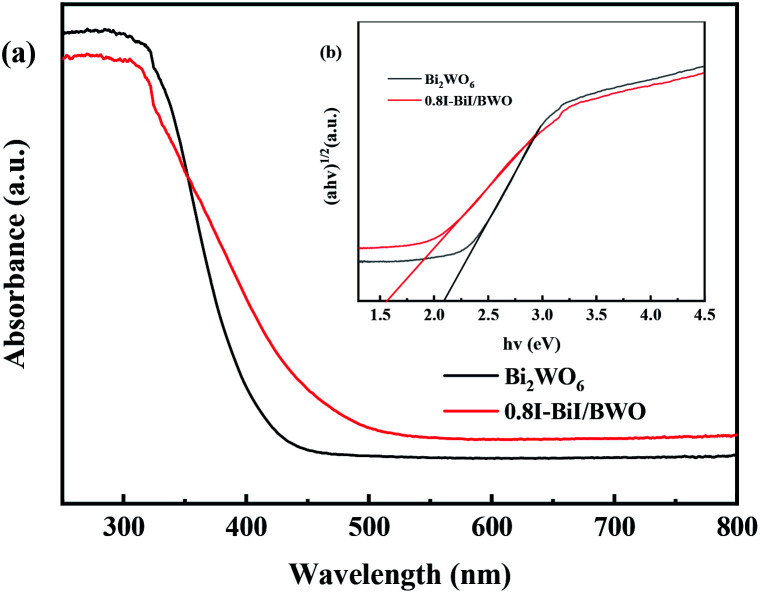
(a) Diffuse reflectance spectra and (b) the Tauc's plots of Bi_2_WO_6_ and 0.8I-BiI/BWO.

Fig. 7(a) depicts the typical UV-vis DRS spectra of pure Bi_2_WO_6_ and 0.8I-BiI/BWO composites. Pure Bi_2_WO_6_ presented the absorption band edge of approximately 415 nm. After KI was added to Bi_2_WO_6_, the absorption band edge was red-shifted, and the absorption band edge of 0.8I-BiI/BWO composites was approximately 480 nm. The 0.8I-BiI/BWO composites increased the absorption edge, which indicated that the prepared sample presented strong visible light response, which was beneficial for increasing its photocatalytic activity.

Moreover, the band gap energy (*E*_g_) of pure Bi_2_WO_6_ and 0.8I-BiI/BWO were calculated using UV-vis DRS data and eqn (2), and the results are presented in Fig. 7(b):^29^

2 *αhν* = *A*(*hν* − *E*_g_)^*n*/2^

where *α*, *h*, *ν*, and *A* are the absorption index, Planck constant, light frequency, and a proportionality constant, respectively. Additionally, *n* is a parameter that depends on the characteristics of the optical transition process in semiconductors. For Bi_2_WO_6_, *n* = 1 for the indirect transition process.^46^ Thus, using the plot of (*αhν*)^1/2^*vs.* (*hν*) (inset of Fig. 7(b)), the *E*_g_ values of pure Bi_2_WO_6_ and 0.8I-BiI/BWO composites were estimated to be 2.1 and 1.6 eV, respectively.

**Fig. 8:**
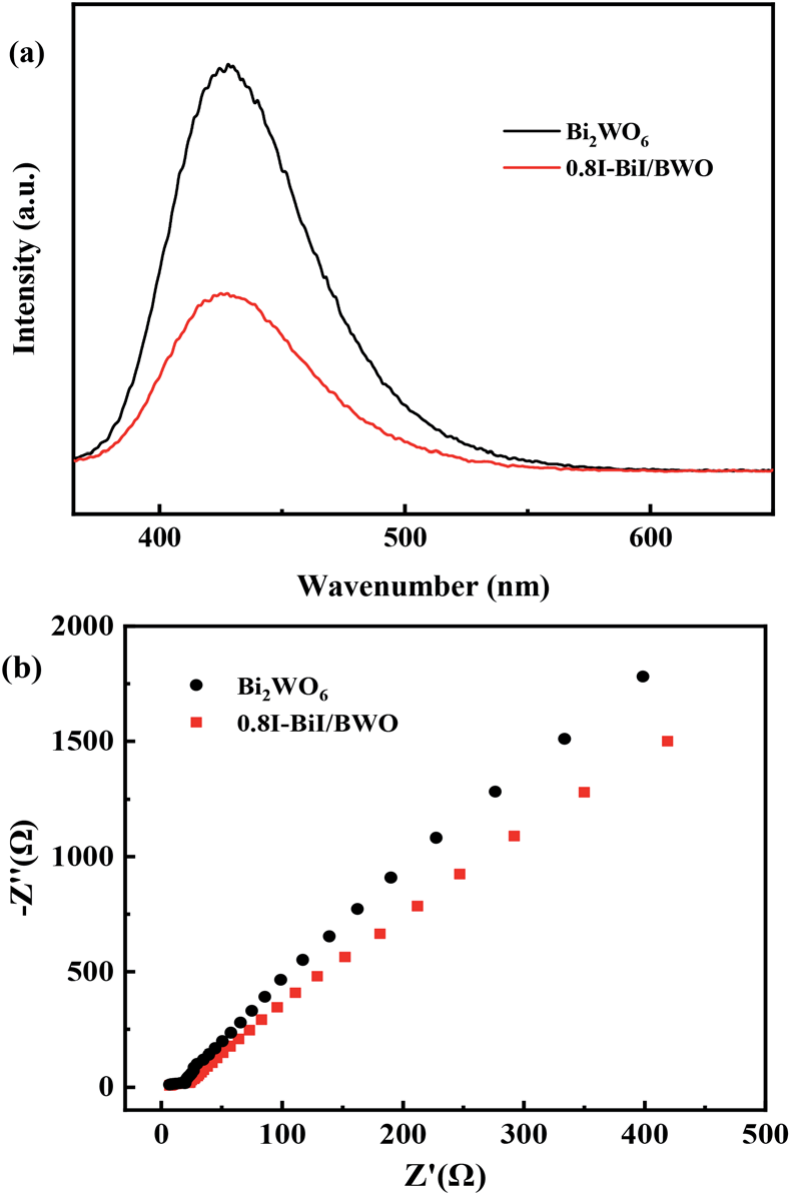
(a) PL spectra and (b) EIS of Bi_2_WO_6_ and 0.8I-BiI/BWO.

The charge separation and electron–hole pair recombination rates of the prepared composites were also studied. Photoluminescence spectroscopy is commonly used to study carrier separation, migration, and recombination. In general, weak emission intensities are associated with low charge carrier recombination rates.^47,48^Fig. 8(a) illustrates the PL spectra of Bi_2_WO_6_ and 0.8I-BiI/BWO composites the excitation wavelength of 365 nm. The PL intensity of 0.8I-BiI/BWO was low, and therefore, it could be concluded that the recombination rate of the internal photogenerated electrons and holes was relatively low. This indicated that the addition of KI to Bi_2_WO_6_ effectively improved the separation ability of the electron–hole pairs, and confirmed that the 0.8I-BiI/BWO composite presented noticeable photoelectric charge migration separation advantages.

The EIS profiles of Bi_2_WO_6_ and 0.8I-BiI/BWO composites are depicted in Fig. 8(b). The semicircle of Bi_2_WO_6_ was larger than that of 0.8I-BiI/BWO, which indicated that the 0.8I-BiI/BWO composite presented lower electrical resistance, more efficient charge separation and electron transfer ability, and higher photocatalytic activity than Bi_2_WO_6_.^49^

### 3.2 Photocatalytic activity

**Fig. 9:**
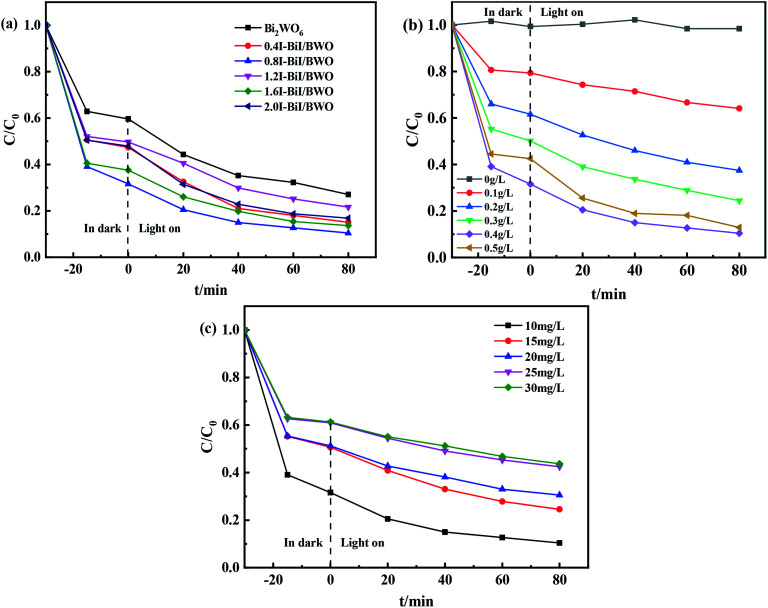
(a) The impact of photocatalytic degradation TC curves, (b) photocatalyst dosages and (c) initial TC concentrations of Bi_2_WO_6_ and 0.8I-BiI/BWO.

The photocatalytic activity of the synthesized 0.8I-BiI/BWO composites was evaluated for the photodegradation of TC aqueous solutions under visible light irradiation. Fig. 9(a) illustrates the time evolution of the UV-vis absorption spectra of TC. Prior to obtaining the UV-vis spectra we performed an adsorption–desorption equilibrium experiment. All samples were able to adsorb TC to some extent. As the irradiation time increased, the absorbance of TC decreased significantly, which indicated that the molecules of TC were decomposed. For the control group, where only the Bi_2_WO_6_ catalyst was added, approximately 73% TC degraded after 80 min of light irradiation. When the 0.8I-BiI/BWO composite was added to the TC solution, the photocatalytic degradation was greatly improved, and TC was completely degraded within 80 min. These results indicated that even if the content of BiI of the BiI/BWO composites was small, the addition of I could greatly improve the degradation ability of the composite photocatalyst for TC. In addition, we determined that 0.8I-BiI/BWO could strongly adsorb TC, which could explain why the adsorption process in the dark improves the visible light degradation ability of the material. The material first achieved the adsorption and enrichment of pollutants, and then the active free radicals on the surface played a major role in the removal of pollutants.^50^

Furthermore, the effect of the catalyst dosage on the degradation of TC was analyzed. When TC was irradiated using a Xe lamp in the absence of photocatalysts, it was barely degraded after 80 min of reaction (Fig. 9(b)), which indicated that the photolysis of TC under visible light irradiation could be neglected. When the dosage of 0.8I-BiI/BWO catalyst was increased from 0 to 0.50 g L^−1^, the degradation rate of TC increased first and then decreased, and the highest degradation rate of approximately 90% was achieved when 0.40 g L^−1^ 0.8I-BiI/BWO catalyst was used. Therefore, the 0.8I-BiI/BWO catalyst affected the photodegradation rate, and 0.40 g L^−1^ was the optimum catalyst dosage. If the dosage of 0.8I-BiI/BWO was too small, the photogenerated electron–hole pairs were too small; consequently, the photon utilization rate was insufficient and the TC molecules could not be completely degraded. If the dosage of the catalyst was too large, not only would the catalyst be wasted, but the costs associated with the process would be increased. Conversely, at high dosage, the catalyst itself could increase the turbidity of the solution or even block photons from reaching the TC solution, and the excess catalyst would reduce the photodegradation rate of TC.^51^ Therefore, considering the cost of the catalyst and the attempts to maximize the use of light energy, 0.40 g L^−1^ was selected as the optimum 0.8I-BiI/BWO dosage for the subsequent experiments.

The effect of the initial concentration of TC on the degradation efficiency of the 0.8I-BiI/BWO catalyst under visible light irradiation was investigated by changing the initial concentration of TC in the range of 10–30 mg L^−1^. The initial concentration of TC significantly affected the photocatalytic degradation reaction. When the initial concentration of TC was 10 mg L^−1^, the degradation rate was 90% after 80 min of irradiation. As the initial TC concentration increased, the degradation rate decreased gradually, and when the initial concentration of TC was 30 mg L^−1^, the degradation rate was 57%. This could be attributed to the increasing amount of TC being able to occupy more active sites of the catalyst, and thereby, slowing the formation of oxidants.^11^ Consequently, the initial TC concentration of 10 mg L^−1^ was selected for the rest of the study.

### 3.3 Identification of degradation intermediates and stability

**Fig. 10:**
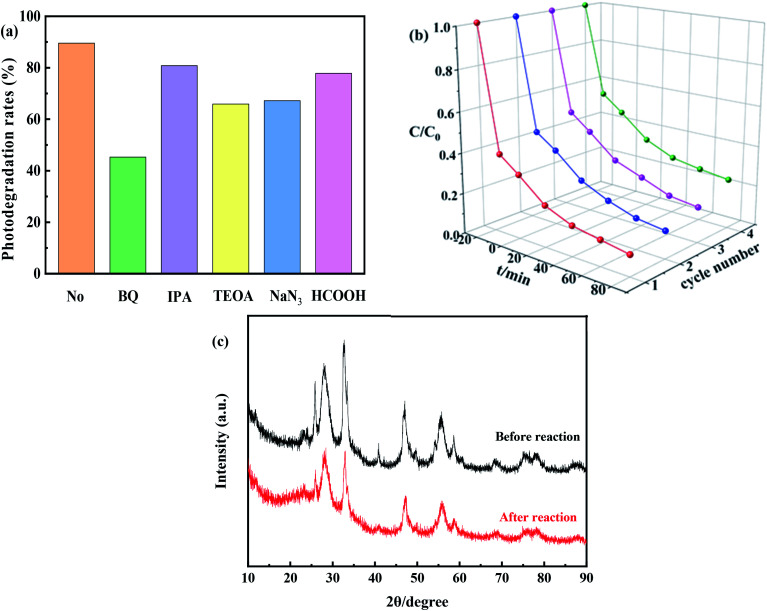
(a) Effect of scavengers on photocatalytic, (b) photocatalytic degradation activity after four cycles of 0.8I-BiI/BWO under simulated sunlight irradiation and (c) XRD patterns of the 0.8I-BiI/BWO before and after the photocatalytic degradation process.

Active species play important roles in photocatalytic reactions. Trapping experiments should be performed to detect the main active species during the photodegradation of TC.^52^ During those trapping experiments, ˙O_2_^−^, ˙OH, h^+^, ˙OH + ^1^O_2_, and e^−^ could be quenched by BQ, IPA, TEOA, NaN_3_ and HCOOH, respectively. The photodegradation efficiency of TC in the absence of trapping agents was 90%, and it decreased to 81% and 67% after the addition of IPA and NaN_3_, respectively, to the reaction system (Fig. 10(a)). These results indicate that the inhibition rate of ^1^O_2_ is 14%, which is negligible.^53^ Conversely, in the presence of TEOA and HCOOH, the photocatalytic degradation efficiencies of TC were 66% and 78%, respectively. This result indicated that h^+^ played a significant role in photocatalytic degradation of TC, likely through a direct electron transfer process. However, when BQ was added to the reaction system, the photocatalytic efficiency of TC decreased significantly to 45%, which indicated that ˙O_2_^−^ was the main active species in the reaction system. And therefore, it could be concluded that the ˙OH and ˙O_2_^−^ species played an important role in the photocatalytic process.

In order to evaluate the cycling performance of 0.8I-BiI/BWO composites, four cycling experiments were performed for the photodegradation of TC. As shown in Fig. 10(b), after four cycles, the photocatalytic degradation activity did not decrease significantly. Furthermore, the XRD patterns of the fresh and used 0.8I-BiI/BWO indicated that the structure of 0.8I-BiI/BWO composites did not change (Fig. 10(c)), and therefore, the 0.8I-BiI/BWO crystals presented good photochemical stability.

### 3.4 Mechanism of photocatalytic activity

**Fig. 11:**
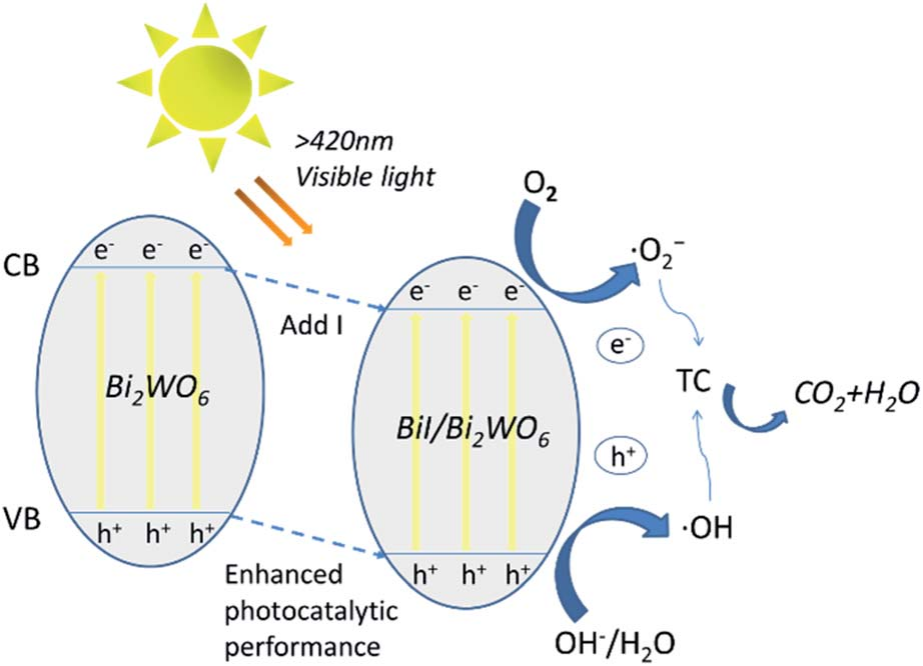
Schematic diagram of photocatalytic enhancement mechanism by BiI/BI_2_WO_6_.

Based on the above results, a possible mechanism for the degradation of TC by 0.8I-BiI/BWO composites has been discussed and analyzed. The introduction of an iodine source initially caused e^−^ in 0.8I-BiI/BWO to be excited from valence band (VB) to conduction band (CB), leaving relatively stable holes in VB, thereby forming electron–hole pairs. During the photoreaction process, TC and 0.8I-BiI/BWO are completely contacted by magnetic stirring. At the same time, e^−^ that move to the surface of the catalyst under internal drive can react with oxygen to form ˙O_2_^−^, and h^+^ react with H_2_O to form ˙OH. Finally, ˙OH and ˙O_2_^−^ initiate a strong oxidation reaction on TC, which is finally oxidized to CO_2_ and H_2_O.^54^ Furthermore, the addition of iodine source to Bi_2_WO_6_ to form 0.8I-BiI/BWO will shifted VB down to enhance the oxidative power of photogenerated holes, thereby improved its photocatalytic activity, and contributed to the degradation of tetracycline in water. All reaction processes as illustrated by the following Fig. 11 and eqn (3)–(6).

3 BiI/BWO + *hν* → h_VB_^+^ + e_CB_^−^

4 e_CB_^−^ + O_2_ → ˙O_2_^−^

5 h_VB_^+^ + H_2_O → ˙OH + H^+^

6 ˙O_2_^−^ + ˙OH + TC → CO_2_ + H_2_O

## 4. Conclusions

In summary, we successfully synthesized BiI/BWO using the facile one-step hydrothermal method. The diffraction peak of BiI could be observed in the XRD pattern of the BiI/BWO composites, which indicated that BiI and Bi_2_WO_6_ successfully formed compounds. In this study, BiI/BWO with different I : W molar ratios were prepared by controlling the amount of KI added to Bi_2_WO_6_. Compared with pure Bi_2_WO_6_, the 0.8I-BiI/BWO composite exhibited excellent performance for the degradation of TC in aqueous media under visible light irradiation, and was able to degrade approximately 90% TC in 80 min. This photocatalytic performance was attributed to factors such as the wide visible light absorption range, high charge separation rate, and large specific surface area of the 0.8I-BiI/BWO composite. This paper not only describes an example of the morphologically dependent photocatalytic activity of Aurivillius-type of oxides, but also provides new ideas for the removal of environmental contaminants using non-metallic supported Bi_2_WO_6_ semiconductor photocatalysts.

## Conflicts of interest

There are no conflicts to declare.

## Supplementary Material
